# Development of a cost-effective, reusable, resuscitative hysterotomy task trainer for emergency medicine trainees

**DOI:** 10.1186/s41077-025-00347-1

**Published:** 2025-03-28

**Authors:** Christiana Agbonghae, Chelsea Rushnell, Brian Lorenzo, John D. Ehlers, Chad Scarboro, Lia Cruz, Sean Fox, Catherine Wares, Christyn Magill, Mark J. Bullard

**Affiliations:** 1 Department of Emergency Medicine, Atrium Health, Carolinas Medical Center, Charlotte, NC USA; 2 ApolloMD, Charlotte, NC USA; 3 Carolinas Simulation Center, Atrium Health, Charlotte, NC USA; 4 Wake Forest School of Medicine, Department of Emergency Medicine, Atrium Health, Carolinas Medical Center, Charlotte, NC USA

## Abstract

We designed, developed, and constructed a reusable, durable, low-cost resuscitative hysterotomy (RH) task trainer with functional and structural fidelity for repetitive practice and education for emergency medicine trainees. The availability of commercial caesarean task trainers is limited, and their high cost often poses a barrier to training. Although similar procedures, RH and traditional caesarean section are unique, and to the authors’ knowledge there are currently no commercial task trainers specifically designed for RH. Current RH literature recommends completing the procedure within five minutes to improve the survival chances of both the fetus and the mother during active or imminent cardiac arrest. While RH is not a technically complex procedure relative to other procedures, it involves specific technical steps and requires clinicians to act decisively. Our RH task trainer was created using low-cost expired materials sourced from our hospital system and additional items purchased online. The RH task trainer was designed to be easily assembled, have minimal recurring material costs, and with quick set-up and clean-up for repetitive practice. When used within a simulation-based scenario, learners are also challenged with the decision to proceed with an RH; thus, providing experiential development of this decision-making step which is unparalleled in comparison to most traditional training formats. Overall, our RH trainer can be built for approximately 230 US dollars. The ability to create low-cost and easily accessible opportunities for repetitive practice of RH contributes to the limited pool of non-commercial RH task trainers, offering valuable experiential instruction for this unique, high-acuity, low-occurrence procedure.

** Brief description** We developed a reusable, durable, low-cost resuscitative hysterotomy (RH) task trainer with functional and structural fidelity for repetitive practice and education for emergency medicine trainees at a tertiary care training hospital. Further, we aimed to create a task trainer that enhanced the cognitive and procedural skills required for performing RH within a high-stress clinical environment.

## Background

Cardiac arrest during pregnancy occurs in approximately 1–2/30,000 maternities [1, 2]. Within the USA, the majority of these patients present to the emergency department [1]; however, globally, RH-appropriate patients may present in different environments [2] and are often cared for by different disciplines including emergency medicine providers, paramedicine, obstetrics, and others. Data on the annual occurrence of RH is limited; however, existing evidence indicates that procedural expediency improves the morbidity and mortality of the mother and fetus [2, 3]. RH is often considered a last resort measure for fetal salvage; however, it offers survival benefits for both the mother during active or imminent cardiac arrest by improving maternal hemodynamics, and for the fetus through concurrent delivery [4]. Current literature recommends completing a RH within five minutes, while some experts have further advocated for an immediate decision to proceed with RH without delay, as soon as a maternal non-shockable rhythm is recognized [4]. Consequently, RH necessitates prompt and decisive action by healthcare providers. Because RH represents a high acuity, low occurrence procedure (HALO), EM trainees do not receive adequate exposure to gain proficiency. In fact, it has been postulated that trainees performing a similar procedure typically need to perform anywhere from 20 to 40 procedures under supervision before competency is achieved [5]. Nonetheless, EM physicians, as well as other disciplines caring for maternal cardiac arrests, must possess the requisite knowledge and technical proficiency to perform RH when treating the appropriate patient. As such, it is imperative that trainees have reusable mechanisms for repetitive practice of RH that allow for the demonstration of procedural steps while in a practice environment that calls for critical decision-making skills. Unfortunately, there are very few commercial caesarean task trainers and their cost can be prohibitive for many academic institutions [6]. Further, to the authors’ knowledge, there are no known commercial task trainers specifically designed for RH on the market.

## Objective

To address the aforementioned challenges, we aimed to design and construct an inexpensive, reusable RH task trainer with functional task alignment for repetitive practice for EM trainees. While recognizing that structural fidelity significantly influences learners’ suspension of disbelief, we prioritized our task trainer design to focus on the “functional correspondence between the simulator and the applied context” [7]. Thus, structural fidelity was important, however not as important as its function within a simulation-based scenario. RH is not a technically complex procedure; arguably, RH requires a few technical steps relative to other procedures. However, the procedure does require experience and critical decision-making skills to act quickly. Our trainer allows learners to (1) gain access into the peritoneum through a midline vertical incision in the abdominal integument; (2) vertically incise the uterine body; (3) avoid incising the bladder; and (4) deliver a term fetus from an amniotic sac. Given the critical importance of the time sensitivity of this procedure and the relative simplicity of the technical steps, our primary goal was to expose learners to the time-critical decision-making process of proceeding with RH while familiarizing themselves with the integral procedural steps. Furthermore, the combination of technical, cognitive, and relational practice affords not only the development of the individual clinician but also lends itself as a modality for readiness practice for all team members caring for a critically ill mother and imminent newborn. Therefore, we designed a reusable RH task trainer that facilitates the practice of the essential procedural steps within an “applied context” [7], ensuring frequent repetition and minimizing the cost of task trainer creation. Our target audience was EM residents; however, we recognize that this RH task trainer could be used by other disciplines within scenarios based on their specific environments.

## Methods

**Fig. 1:**
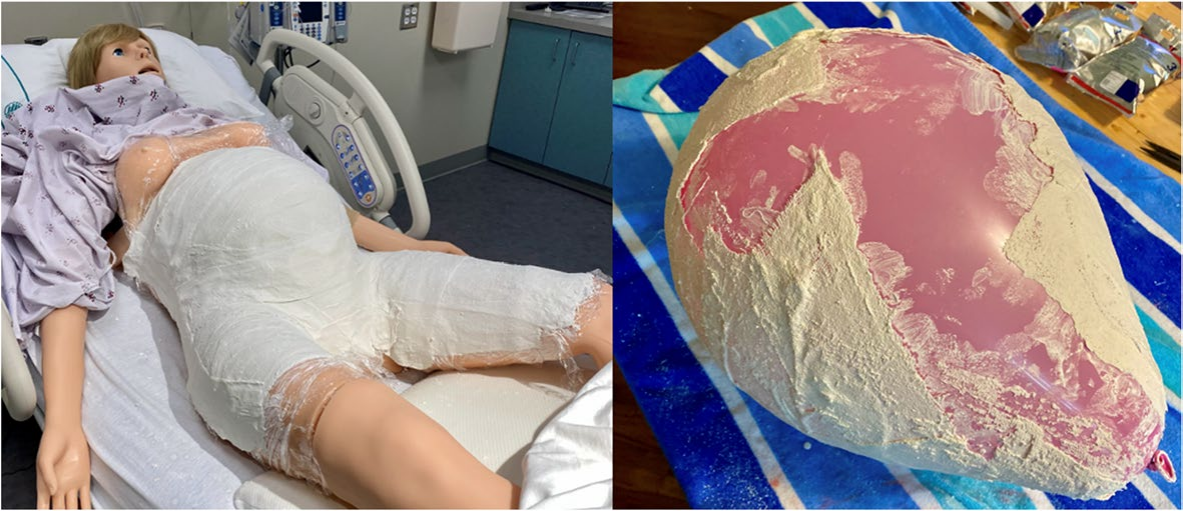
Plaster casting molded to Victoria birthing simulator and balloon for uterus creation

**Fig. 2:**
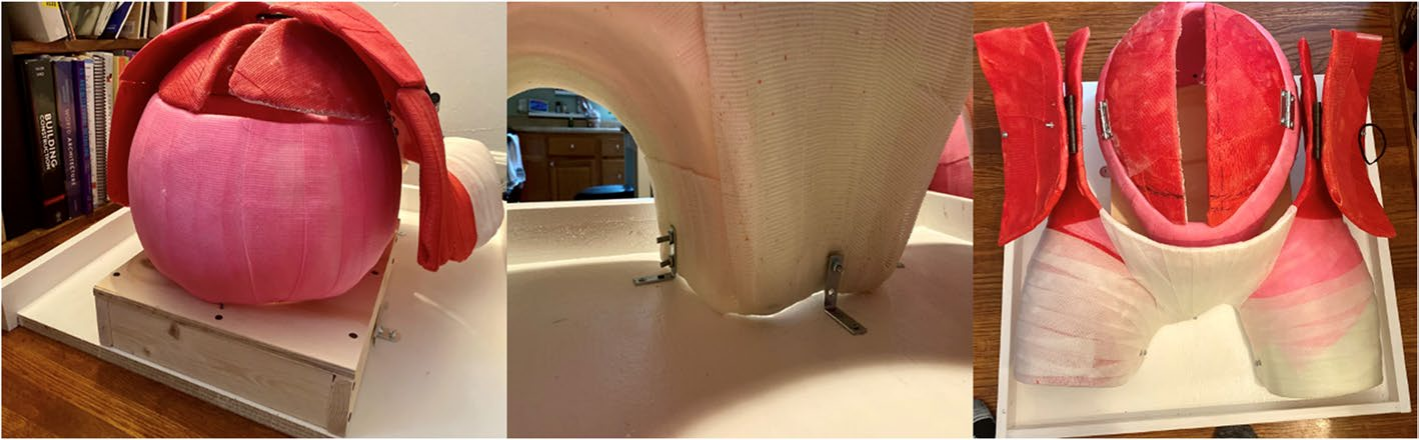
Fiberglass RH trainer mounted to plywood structure

**Fig. 3:**
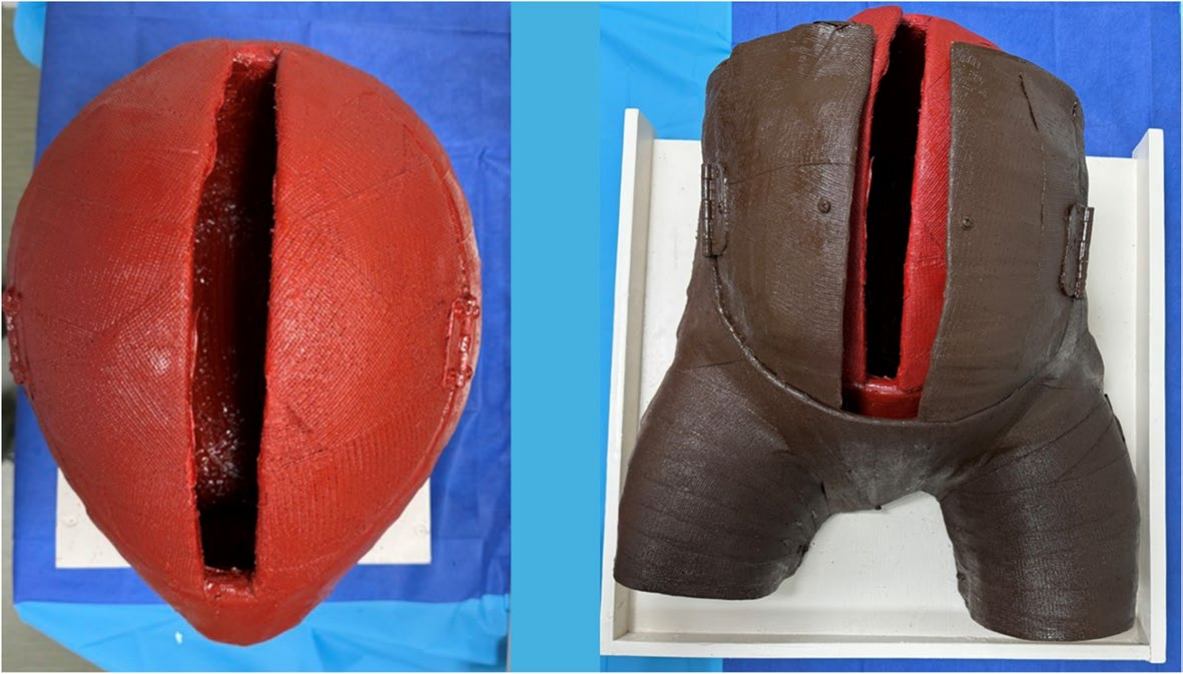
Painted fiberglass uterus and abdomen mounted on painted plywood

**Fig. 4:**
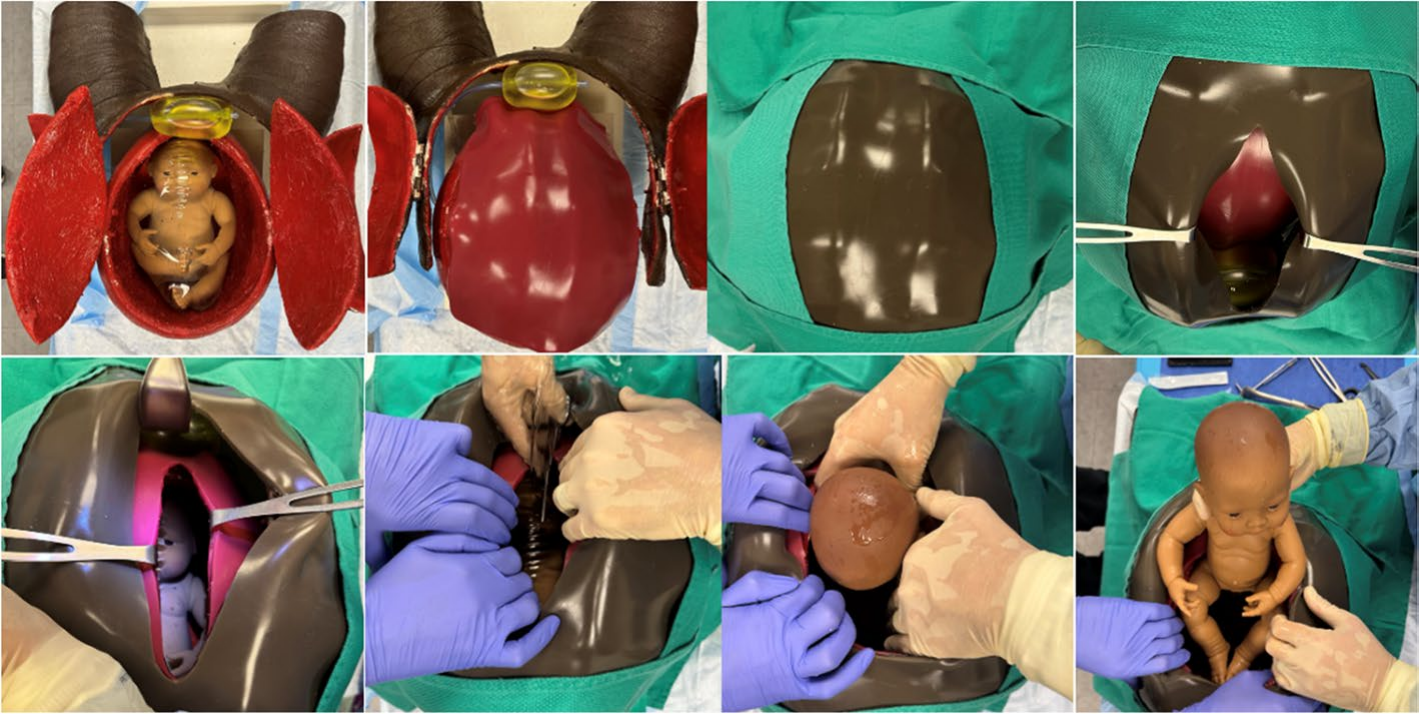
RH task trainer: amniotic sac with Bakri balloon as bladder in use

The RH task trainer we developed integrated the specified materials outlined in Table 1. The RH task trainer was created using low-cost expired materials sourced from our hospital system and through online vendors, ensuring accessibility for educators. Additionally, it was intended to be constructed with minimal recurring operating costs and facilitates quick set-up and clean-up for repetitive practice sessions. The trainer was made using our simulation center’s Victoria S2200 birthing simulator (Gaumard Scientific, Miami, FL, USA) as an abdominal mold. Plastic wrap was placed over Victoria’s abdomen and upper legs to protect the manikin. Casting plaster was then draped over the same area and allowed to harden. Additionally, a balloon was used in a similar fashion to create a plaster mold for the uterus (Fig. 1). Delta-Lite Plus fiberglass casting material (BSN Medical, Charlotte, NC, USA) was draped over the respective plaster molds to form the finalized structure of the task trainer to ensure durability with repetitive exposure to liquid. The fiberglass uterus and abdomen were then secured to ¾” plywood wooden platforms with L-brackets for stability. The anterior abdominal wall, as well as the uterus, were respectively fitted with hinges to create an area for a large midline incision and retraction of the integument and uterine musculature (Fig. 2). The interior of the uterus was coated with boat sealant (Loctite, Rocky Hill, CT, USA) to further waterproof the uterine cavity. The fiberglass structure was then painted to approximate skin and muscle coloration. Incisable skin and uterine muscle were created at our simulation center using a 1:1 ratio of Smooth-On Eco-Flex and Eco-Flex Gel (Smooth-on Inc., Macungie, PA, USA) and colored to match the fiberglass trainer. An amniotic sac was created using a large thermoplastic polyurethane balloon (Dilcamrita, Seattle, WA, USA) filled with water and placed around a simulated, term vinyl doll (Miniland Educational Corp, Miami, FL, USA). An anatomically correct newborn doll was purchased and used as a term fetus due to its minimal expense and its vinyl construction which allowed for repetitive placement in a liquid environment (amniotic sac). A Bakri balloon (Cook Medical, Bloomington, IN, USA) was inflated with water and attached to the interior caudal aspect of the peritoneum to represent the bladder (Figs. 3 and 4).

**Table 1:** Itemized materials for emergency RH trainer and cost

Product title	Quantity	Total cost (USD)
Miniland Educational–15.75′′ Anatomically Correct Newborn Baby Doll, Hispanic Girl	1	32.98
Dilcamrita 26′′ Large-mouth thermoplastic polyurethane balloons	15	12.99
¾” plywood (4′ × 8′)	1	65.00
3.5′′ × 5/8′′ radius hinges	2	7.56
3′′ hinges	2	2.48
3′′ × 0.75′′ L-brackets	4	8.58
Loctite PL Marine Fast Cure Adhesive Sealants, 20 Oz	2	34.96
Krylon Fusion All-In-One Espresso Paint & Primer Spray Paint Gloss, 12 oz	2	13.96
Krylon Fusion All-In-One Red Pepper Paint & Primer Spray Paint Gloss	2	15.96
Loctite Clear Silicone Waterproof Sealant, Single Tube, 2.7 oz	1	7.99
Smooth-On Eco Flex and Eco-Flex Gel abdominal skin/uterine muscle	1 each	27.00
Bakri balloon (expired from hospital)	1	0.00
Plaster casting material (expired from hospital)	~ to cover manikin	0.00
Delta-Lite Plus fiberglass casting material (expired from hospital)	~ 20 rolls	0.00
**Total creation costs: USD (2024)**		**$229.46 USD**
**Total recurring costs (iterative use): USD (2024)**		**$28.00 USD**

## Discussion

This model represents a cost-effective, easily created, durable, and reusable RH task trainer with high functional fidelity and quality physical resemblance. Despite its procedural simplicity, RH entails a crucial cognitive leap for clinicians, given its time-sensitive nature and significant impact on patient outcomes. The attainment and retention of RH skills necessitate repetitive practice in both cognitive and procedural skill domains, as evidenced by decreased incision-to-delivery times observed in traditional caesarean section learning curves [5].

Our RH task trainer focuses on the necessary step-by-step functional tasks essential for RH performance. Other non-commercial task trainers have demonstrated improved learner confidence in RH [8, 9], enhanced knowledge [10], improved performance in procedural steps [11], and enhanced learning compared to more traditional lectures and reading alone [12]. When used within a human-patient manikin simulation-based scenario, this RH trainer further adds to the experiential fidelity by requiring the learner to make the challenging time-dependent decision to proceed with RH.

Commercial trainers are limited in number and are cost-prohibitive for many simulation programs [6, 11, 13]. Commercial trainers often place excessive emphasis on high physical resemblance leading to the belief that more structurally accurate trainers are of higher educational quality. High physical resemblance, however, can exponentially add to higher costs found in commercial task trainers. Further, it has been postulated that the overemphasis on structural fidelity can actually reduce the educational effectiveness of a simulator [7]. When design emphasis focuses on the functional aspects of a trainer used within a more global simulation context, learning should not be affected [7]. Thus, non-commercial trainers focusing on function can be created without exponential costs and without negatively affecting learning [7].

With no known commercially available task trainers designed specifically for RH, educators would do well to create non-commercial task trainers focused on function, cost, durability, limited recurrent purchases, limited iterative build-time, and ease of use (including set up and clean up). All must be considered for successful repetitive training with non-commercial task trainers. The authors easily created this RH task trainer with accessible, inexpensive products, with quick set-up, minimal recurring costs, and quick clean-up for rapid succession of deliberate practice of RH.

While several non-commercial RH trainers have been described in the literature, many entail recurrent uterine creation and recurring costs for repetitive practice [6, 8, 12]. Our model design was explicit in using materials such as a fiberglass shell and a completely vinyl infant for durability within a liquid environment. We believe that the simplicity and limited time preparing for frequent repetitive use (simply refilling two balloons with tap water and placing two skins on the uterus and abdomen, respectively) outweigh the complexity of this trainer’s initial build. Assuming abdominal skin and uterus myofascial replacements are made ahead of time, it takes approximately five minutes to clean up; replace the amniotic sac and bladder, and replace the skin prior to the next use.

Other RH trainers have used animal products to simulate anatomic structures within their trainers [6, 14], adding clean-up time and the need to purchase and dispose of animal products. Some trainers also require repetitive creation of the uterus with fabric, yoga mats, jelly, colored fluid, duct tape, etc. [6, 9, 15]; all of which add to recurring creation time and costs. Further, some non-commercial RH trainers have required additional purchases such as a 3D printer [16] or outsourced design product experts to build the trainer rather than being built by the educators [10]. Our RH trainer’s only recurring costs are integument and uterine myometrium created at our simulation center (approximately 27 US dollars) and balloon repurchasing (approximately 1 US dollar/balloon) for representation of the amniotic sac. The bladder was simulated using an expired Bakri balloon at no cost from our hospital system. However, during simulation-based education using this task trainer, it was found that many learners incised the “bladder” during the abdominal incision requiring the replacement of the Bakri balloon. The bladder could be simulated by a latex balloon if expired Bakri balloons were not available for use. The limited, low-cost recurring materials, lack of animal product, and simplicity of design allow our RH trainer to be used repeatedly at a fraction of the cost of commercial traditional caesarean trainers with easy step-up and clean-up compared to other published non-commercial designs.

RH remains an essential competency for EM physicians to develop and maintain despite perhaps never having to perform this procedure during their entire careers. Our RH trainer stands out for its simplicity, accessibility, durability, and affordability, contributing to the collection of non-commercial trainers for experiential instruction in this critical, albeit infrequent, emergency procedure essential for healthcare teams managing maternal cardiac arrests. This ability to affordably and readily create opportunities for repetitive practice of RH contributes to a nascent collection of non-commercial RH task trainers. These resources are invaluable for experiential instruction in managing this distinctive, high-acuity, low-occurrence emergency procedure.

## Data Availability

No datasets were generated or analysed during the current study.
